# Other PHAC publications

**DOI:** 10.24095/hpcdp.46.3.07

**Published:** 2026-03

**Researchers from the Public Health Agency of Canada also contribute to work published in other journals and books. Look for the following articles published in 2024 and 2025:**

Afifi TO, Fortier J, Salmon S, Taillieu TL, Osorio A, Roos L, […] **Tonmyr L**, et al. Youth COVID-19 stressors and associations with self-perceived health, depression, anxiety, and at-risk alcohol and cannabis use. Facets. 2024;9(1):1-10. https://doi.org/10.1139/facets-2023-0160

Collins E, **Al-Jaishi A, Farrow A, Amankwah N**, Georgiades S, Salt M, […] **Holmes K, Edjoc R**. Household income among families with autistic children and youths in Canada: a cross-sectional matched cohort study. BMJ Open. 2025;15(11):e096019. https://doi.org/10.1136/bmjopen-2024-096019

**de Rubeis V, Tonmyr L**, Rahman S, **Pagaduan J, Drysdale M, Morissette K**, […] **Aylward E, Nanziba F, Powell S, Corrin T, Khan A, Boland LS**. Changes in child maltreatment occurrence during the COVID-19 pandemic: a systematic review. Child Abuse Negl. 2025;169(Pt 1):107744. https://doi.org/10.1016/j.chiabu.2025.107744

Duggan L, **Lang JJ**, Timmons BW, Tucker P, Chaput J-P. Adherence to 24-hour movement guidelines by long-term health condition in Canadian children and youth. J Epidemiol Popul Health. 2025;73(5):203149. https://doi.org/10.1016/j.jeph.2025.203149

Lang E, **Gray C**, LeBlanc JC, Colquhoun H, **Traversy G**. Recommendation on screening adults for depression using a screening tool. CMAJ. 2025;197(35):E1132-43. https://doi.org/10.1503/cmaj.250237

Lunny C, Jain N, Nazari T, Kosaner Kliess M, Santos L, Goodman I, […] **Stevens A**, et al. Exploring the methodological quality and risk of bias in 200 systematic reviews: a comparative study of ROBIS and AMSTAR-2 tools. Res Synth Methods. 2026;17(1):63-92. https://doi.org/10.1017/rsm.2025.10032

**McKinnon B, Hovdestad W, Campeau A, Pollock N**, Afifi TO, Gonzalez A, […] **Tonmyr L**. Childhood abuse prevalence in Canada: insights from six national surveys. Child Indic Res. 2025. https://doi.org/10.1007/s12187-025-10302-1

**Prince SA, Roberts KC, Betancourt MT**, Colley RC. Reflections on daily steps and health outcomes. Lancet Public Health. 2025; 10(11):e901. https://doi.org/10.1016/S2468-2667(25)00247-6

Shahidi FV, Andreacchi AT, Fuller AE, **Blair A**, Carnide N, Harris MA, et al. Employment quality and mortality in Canada. J Epidemiol Community Health. 2025;80(1):27-34. https://doi.org/10.1136/jech-2025-224434

Taillieu TL, Salmon S, Fortier J, Stewart-Tufescu A, Osorio A, MacMillan HL, […] **Tonmyr L**, et al. Cross-sectional and longitudinal associations between adolescent vaping and physical and mental health problems. Addict Behav Rep. 2025;22:100633. https://doi.org/10.1016/j.abrep.2025.100633

Veroniki AA, Tricco AC, Rangira D, McKenzie JE, Li T, Straus SE, […] **Stevens A**, et al. Updating the PRISMA reporting guideline for network meta-analysis: a scoping review. J Clin Epidemiol. 2025;188:111985. https://doi.org/10.1016/j.jclinepi.2025.111985

